# Superconductivity in a uranium containing high entropy alloy

**DOI:** 10.1038/s41598-020-61666-z

**Published:** 2020-03-13

**Authors:** W. L. Nelson, A. T. Chemey, M. Hertz, E. Choi, D. E. Graf, S. Latturner, T. E. Albrecht-Schmitt, K. Wei, R. E. Baumbach

**Affiliations:** 1 National High Magnetic Field Laboratory, Florida State University, Tallahassee, FL 32310 USA; 2 Department of Physics, Florida State University, Tallahassee, FL 32306 USA; 3 Department of Chemistry, Florida State University, Tallahassee, FL 32306 USA

**Keywords:** Physics, Condensed-matter physics, Electronic properties and materials, Structure of solids and liquids, Superconducting properties and materials

## Abstract

High entropy alloys (HEA) are an unusual class of materials where mixtures of elements are stochastically arrayed on a simple crystalline lattice. These systems exhibit remarkable functionality, often along several distinct axes: e.g., the examples [TaNb]_1-x_(TiZrHf)_x_ are high strength and damage resistant refractory metals that also exhibit superconductivity with large upper critical fields. Here we report the discovery of an *f*-electron containing HEA, [TaNb]_0.31_(TiUHf)_0.69_, which is the first to include an actinide ion. Similar to the Zr-analogue, this material crystallizes in a body-centered cubic lattice with the lattice constant *a* = 3.41(1) Å and exhibits phonon mediated superconductivity with a transition temperatures *T*_c_ ≈ 3.2 K and upper critical fields *H*_c2_ ≈ 6.4 T. These results expand this class of materials to include actinide elements, shows that superconductivity is robust in this sub-group, and opens the path towards leveraging HEAs as functional waste forms for a variety of radioisotopes.

## Introduction

High entropy alloys (HEA) are multicomponent mixtures of randomly combined elements where, instead of producing disordered mixtures of lower order crystalline compounds (binaries, ternaries, etc.), solid solutions with simple structures (e.g., body center cubic, face center cubic, or hexagonal close packed) are formed^1–5^. This occurs when the mixing entropy overrides simple stoichiometric/crystalline phase selection, and leads to a large chemical compositional space that is distinct from that of traditional alloys where a few molar percent of one element is added to another (e.g., as for sterling silver, modern steels, and Be-Cu). Many attractive behaviors are observed in HEAs, including fracture resilience at cryogenic temperatures^6,7^, excellent mechanical properties at elevated temperatures^8^, high strength^9,10^, and high resistance to radiation damage^11–14^. An emerging class of HEAs are those hosting 4d and 5d transition metals that exhibit phonon mediated superconductivity with large upper critical fields^5,15,16^. Importantly, many HEAs are functional in multiple distinct ways, making them attractive as candidates for a variety of applications; e.g., as next generation wires for superconducting magnets or for use under extreme conditions^1–5^.

Most studies have focused on combinations of transition metal elements to produce HEAs, but it is interesting to ask whether lanthanide or actinide elements can be introduced as well. This would have several advantages with both practical and basic science implications. For instance, *f*-electron elements such Ce, Yb, U, Np, Pu, and Am exhibit valence flexibility, have a tendency to hybridize with conduction electrons and have large spin anisotropy^17^. Other examples such as Nd, Sm, Gd-Tm, and Cm-Cf feature well localized *f*-states with associated magnetism^18^. These characteristics often lead to novel properties that include unconventional magnetic order, complex electronic order, topologically protected states, structural instabilities, and superconductivity^19–21^. Indeed, recent studies of HEAs that are composed entirely of lanthanide elements show that they exhibit complex magnetism with evidence for spin-glass behavior^22–24^. It was also recently shown that the presence of a high entropy alloy-type blocking in the series REO_0.5_F_0.5_BiS_2_ enhances the superconductivity of this system^25^. Actinide based alloys are also of interest for development of solid solutions (i) that form under extreme conditions, (ii) as radiation resistant cladding and robust waste forms, or (iii) for long term storage of hazardous radioisotopes.

Here we report results for the HEA system [TaNb]_0.31_(TiUHf)_0.69_, which is a variant of the alloy series [TaNb]_1-x_(TiZrHf)_x_^5,15,16^. To our knowledge, this is the first examples of an actinide containing HEA, but point out that its existence is readily anticipated given that in many cases zirconium and uranium can be considered as chemical and electronic analogues to each other; they both can adopt a tetravalent electronic configuration and have similar atomic sizes. Therefore, we expect that this will merely be the first of many such examples and that other lanthanides and actinides with multi-configurational valences (e.g., Ce, Np and Pu) will readily be introduced into such environments. Similar to the Zr analogues, X-Ray diffraction measurements show that this material crystallizes in the cubic space group *Im*$\bar{3}$*m* and occupies a body-centered cubic lattice (BCC) with the lattice constant *a* = 3.41(1) Å. Elemental mapping using energy dispersive spectroscopy shows a uniform distribution of the elements throughout the arc-melted samples. The alloy is difficult to abrade or cut, is readily cold worked into thin plate, and is resistant to many destructive environments including concentrated acids. We also show that, similar to the Zr containing analogues, the material exhibits bulk superconductivity with an onset transition temperatures *T*_c_ ≈ 3.2 K and large upper critical field *H*_c2_ ≈ 6.4 T, exceeding what is seen for most uranium based superconductors. Like other HEA superconductors, this occurs in a highly disordered environment which likely provides pinning centers for superconducting vortices. Bulk electrical transport, magnetization, heat capacity, thermopower, and thermal conductivity measurements reveal other similarities to the Zr-analogues, where we find that the *f*-electrons are delocalized and that the chemical disorder results in a large and nearly temperature independent electrical resistivity and a small thermal conductivity. [TaNb]_0.31_(TiUHf)_0.69_ thus emerges as a representative of what likely is a much larger family of actinide materials that would be of importance for both technological (e.g., development of durable waste forms) and basic science (reservoir for superconductivity) reasons.

## Results

### Structural and chemical characterization of [TaNb]_0.31_(TiUHf)_0.69_

**Figure 1:**
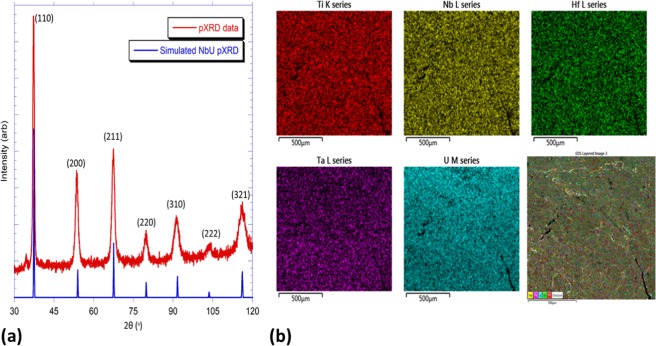
Summary of structural and chemical data for [TaNb]_0.31_(TiUHf)_0.69_. (**a**) X-ray diffraction pattern for the as-cast [TaNb]_0.31_(TiUHf)_0.69_. The pattern was collected for a small piece removed from the very hard alloy, which was impossible to reduce to a powder even when cooled using liquid nitrogen. The pattern is isomorphous with a series of Group IV/V alloys such as NbU^20^, HfTa^21^, TaTi^22^, and NbZr^23^ and related “type A” HEAs^5^, which form in the Cubic space group *Im*$\bar{3}$*m* and occupy a body-centered cubic lattice (BCC). The unit cell axis length is calculated to be 3.41(1) Å. (**b**) Energy Dispersive Spectroscopy data for an as-cast piece of [TaNb]_0.31_(TiUHf)_0.69_. These data were used to identify inhomogeneity in the sample and quantify the apparent elements. Elemental mapping of both the flattened sample and chips cut from the bulk indicated a homogenous mixture of Ti, Nb, Hf, Ta, and U, with no enriched zones.

Polycrystalline specimens were produced using the arc furnace method. The resulting boules are difficult to abrade, cut, or reduce to a granular form, which required that powder X-Ray diffraction measurements be performed on pieces that were pounded flat in liquid nitrogen. The resulting diffraction pattern (Fig. 1a) is isomorphous with a series of Group IV/V alloys such as NbU^26^, HfTa^27^, TaTi^28^, and NbZr^29^ and related “type A” HEAs^5^. All of these alloys form in the Cubic space group *Im*$\bar{3}$*m* and occupy a body-centered cubic lattice (BCC). These results are consistent with the literature, and the notation of Group V metals given in brackets and Group IV metals given in parentheses is maintained from ref. ^5^. The unit cell axis length is determined to be 3.41(1) Å, which is slightly larger than the NbU analogue employed for powder diffraction comparisons, and at the upper end of the range observed for an alloy which employed Zr instead of U. There is some indication of an impurity phase, likely uranium, as indicated by a weak peak at 2θ = 34.5°. However, this peak is so small as to be unquantifiable. As noted previously, the reflections are exceptionally broad (FWHM 2–3% of peak position) due to disorder in the HEA and inherent limitations of powder diffraction on a single block. Energy Dispersive Spectroscopy was used to identify inhomogeneity in the sample and quantify the elements (Fig. 1b). Elemental mapping of both the flattened sample and chips cut from the bulk indicated a homogenous mixture of Ti, Nb, Hf, Ta, and U, with no enriched zones. While standard-less quantification is only approximate, we obtain the stoichiometries listed in Table S1. These data were calculated from 29 spectra on a flat polycrystalline surface, and there was no statistical difference between quantification on the flat surface or on small chips. Phase matching found no minor phases to be present when a surface approximately 4 mm^2^ was examined. The deficiency in Nb and Ta is persistent, whether the quantification was on the flat surface or razor-cut chips off the bulk product. Therefore, we conclude that the product is approximately [TaNb]_0.31_(TiUHf)_0.69_.

In order to assess the chemical stability of the alloy it was immersed in several acidic solutions for a period of two minutes and observed for evidence of degradation or chemical reactivity. The sample was thoroughly rinsed with deionized water after each reactivity test. We find that it does not immediately dissolve in: nitric acid at 0.001 M, 1 M, 3 M, 6.5 M, or concentrated (16 M); 5 M hydrochloric acid; 6 M sulfuric acid; 9 M hydrobromic acid; aqua regia; or 9 M perchloric acid. It does dissolve readily in 29 M hydrofluoric acid. This is in agreement with earlier results for other HEAs; for instance, Al_0.5_CoCrCuFeNiB_*x*_ was reported to be more resistant to corrosion than stainless steel in a 1 N sulfuric acid solution^30^.

### Superconductivity in [TaNb]_0.31_(TiUHf)_0.69_

**Figure 2:**
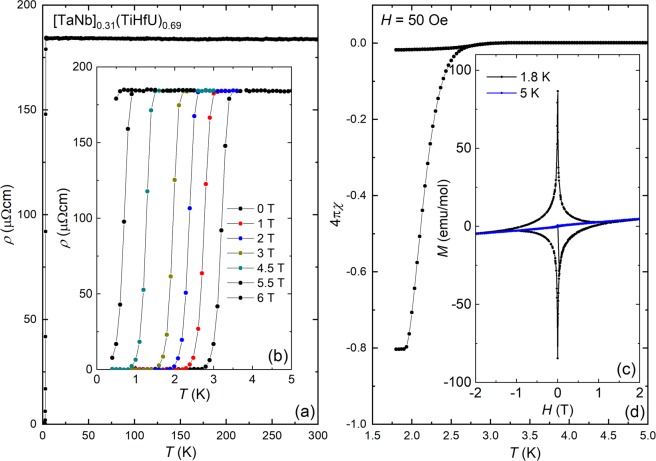
Summary of electrical transport and magnetization data for [TaNb]_0.31_(TiUHf)_0.69_. (**a**) Electrical resistivity *ρ* between the temperatures *T* = 2–300 K for [TaNb]_0.31_(TiHfU)_0.69_. (**b**) Magnetic field *H* dependence of *ρ*(*T*) for *H* = 0–6 T, focusing on the superconducting phase transition *T*_c_. (**c**) DC magnetization data collected in an applied magnetic field of *H* = 50 Oe showing both the zero field cooled and field cooled temperature *T* dependence, which reveal bulk superconductivity. (**d**) Magnetization *M* vs. *H* loop at temperatures *T* = 1.8 and 5 K. For *T* = 1.8 K, the behavior is consistent with expectations for a type 2 superconductor where magnetic flux penetrates the bulk of the sample. At 5 K the M(H) curve is consistent with expectations for a Pauli paramagnet where the U *f*-states are delocalized. DC magnetic susceptibility *χ* = *M*/*H* collected in *H* = 0.5 T for *T* = 1.8–300 K are shown in Fig. S1, where the weak temperature dependence and lack of Curie-Weiss behavior also indicate that the uranium *f*- electrons are not well localized.

The temperature dependent electrical resistivity data are presented in Fig. 2a,b, where it is seen that *ρ*(*T*) increases slowly with decreasing temperature to reach a residual resistivity value near *ρ*_0_ = 183 μΩcm. Below *T*_c_ ≈ 3.2 K there is an abrupt drop to a zero resistance state which indicates the onset of superconductivity. As seen in the inset, the transition has a width of approximately Δ = 0.4 K, and together with the large *ρ*_0_ this suggests that the chemical disorder impacts both the normal state behavior and superconductivity. Similar behavior was earlier seen for the Zr analogues^16^, although the value of *ρ*_0_ for the U case is somewhat increased by comparison. The magnetic field dependence of the transition is shown in the inset, where *T*_c_ is gradually suppressed with increasing *H* and is fully suppressed for values above 6 T. We also examined the pressure dependence of *T*_c_ (Fig. S1), where we find that it almost completely insensitive to hydrostatic pressure for *P* < 2.3 GPa. This is like what is seen for other HEA superconductors, where *T*_c_ remains nearly constant up to hundreds of GPa^5,31^.

The magnetic susceptibility *χ*(*T*) data measured in a field of 50 Oe for both field cooled and zero field cooled conditions are shown in Fig. 2c,d. Below about *T*_c_ ≈ 2.5 K the ZFC curve shows a strong diamagnetic response due to the Meissner effect, which yields almost the ideal diamagnetic value −4πχ that is expected for a bulk superconductor. For the FC curve the small recovery of the diamagnetic signal may indicate that the material exhibits strong vortex pinning. Isothermal magnetization *M*(*H*) curves for 1.8 and 5 K are shown in Fig. 2c. Here, the linear response of the Meissner state at low fields is rapidly followed by a decrease in the magnitude of *M* with increasing *H*, as is expected due to flux penetration in the vortex state for a type-II superconductor. The *χ*(*T*) and *M*(*H*) data in the normal state (Figs. S2 and 2c) indicate that the uranium *f*-electrons are not well localized in this system. In particular, *χ*(*T*) is not described by a Curie-Weiss law and instead is weakly temperature dependent, while *M*(*H*) for *T* > *T*_c_ shows a weak linear response.

**Figure 3:**
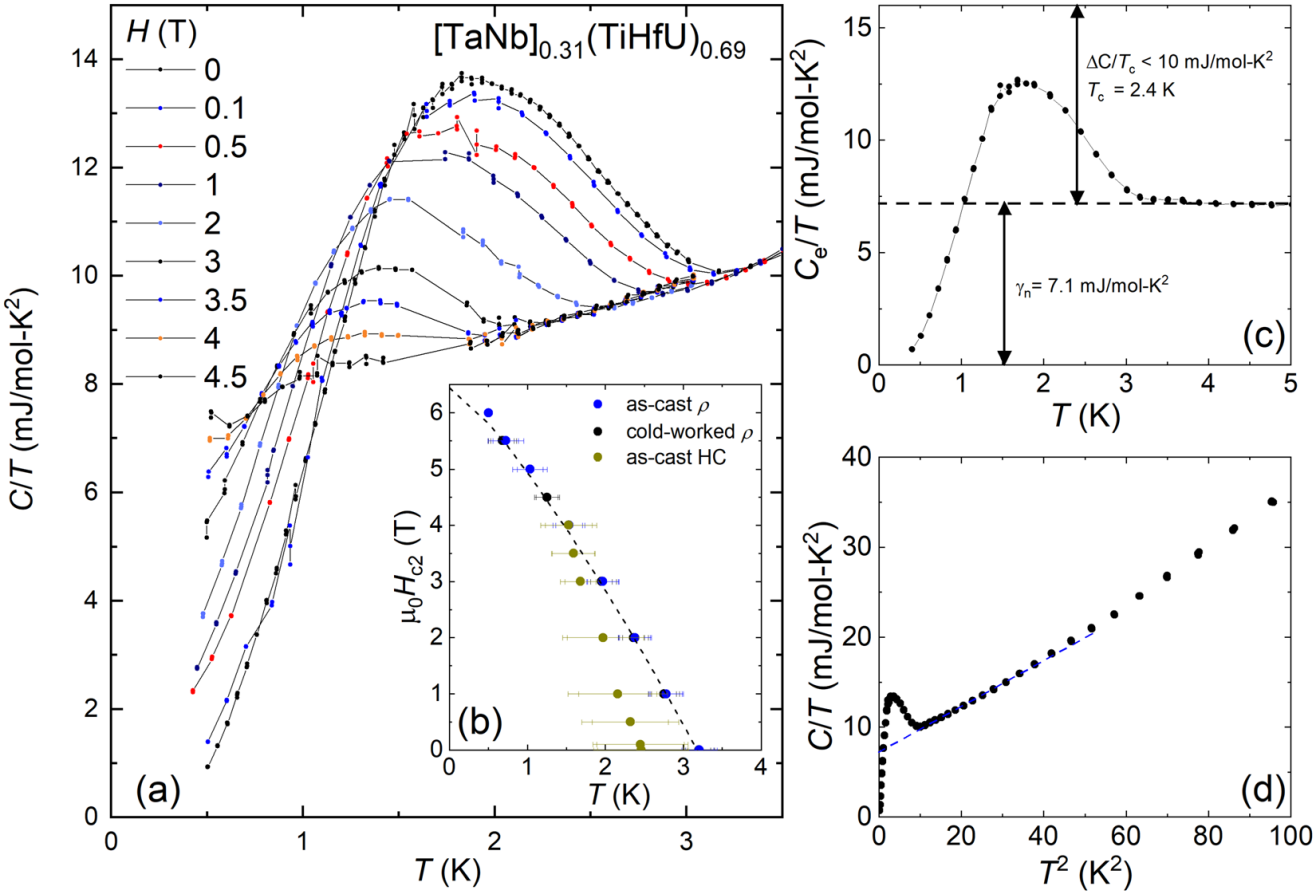
Heat capacity data for [TaNb]_0.31_(TiUHf)_0.69_. (**a**) Temperature dependent heat capacity measurements for temperatures *T* = 0.5–3.5 K and magnetic fields *H* = 0–4.5 T. The bulk superconducting phase transition at *T*_c_ is seen as a broadened lambda-like peak that is suppressed with increasing *H*. (**b**) The upper critical field *H*_c2_ vs *T* defined both from the lambda feature in the heat capacity and as the 50% value of the electrical resistivity (see Fig. 2). Results are shown both for an as-cast sample and for a piece that was cold worked under liquid nitrogen into a thin sheet. The dotted line is a fit to the data, as described in the text. (**c**) The electric contribution to the heat capacity divided by *T*, where the phonon contribution (shown in panel d as a dotted line) has been subtracted. The electronic coefficient of the heat capacity *γ* and the size of the jump Δ*C*/*T*_c_ are shown. (**d**) *C/T* vs. *T*^2^ showing that over a broad *T* range the data are described by the expression *γ* + *βT*^2^.

The heat capacity divided by temperature *C*/*T* data for temperatures spanning *T*_c_ and for magnetic fields between 0–4.5 T are summarized in Fig. 3a. For *H* = 0, the superconducting phase transition appears as a broadened lambda-shaped feature where *T*_c_ = 2.4 K is defined as the midpoint of the rise in *C*/*T* (Fig. 3c), although its onset is found at *T*_c,onset_ = 3.2 K. The size of the jump Δ*C*/*T*_c_ < 10 mJ/mol-K^2^ is estimated using an equal entropy construction, but the broadness of the peak prevents determination of a definitive value. As shown in Fig. 3d, a fit to the data for *T* > *T*_c_ using the expression *γ* + *βT*^2^ yield the values *γ* = 7.1 mJ/mol-K^2^ and *β* = 0.26 mJ/mol-K^4^ and from this we estimate that Δ*C*/*γ T*_c_ is slightly less than 1.43, consistent with expectations from the BCS theory for a phonon-mediated superconductor in the weak electron-phonon coupling limit. We also calculate a Debye temperature near *Θ*_D_ = (12π^4^*R*/5*β*)^1/3^ = 193 K, where *R* is the ideal gas constant. Within the superconducting state *C/T* extrapolates towards zero, suggesting that the gap is isotropic and does not include nodes. This is in contrast to what is seen for some other strongly correlated *f*-electron superconductors with likely non-s-wave superconductivity^19^ but similar to results for the Zr containing HEA analogues^5,16^. In order to assess the gap size, we consider the BCS expression 2Δ(0)/k_B_*T*_c_ = 3.52, from which we estimate Δ(0) ≈ 0.36 meV. Taken together, these results reveal a close similarity to the Zr-based analogues^15,16^.

In Fig. 3b we show the temperature dependence of the upper critical field curve *H*_c2_ determined from both electrical resistivity and heat capacity measurements. We also compare results from electrical transport studies of both an as-cast specimen and one that was cold worked into a flattened plate, where there is no discernable change in the behavior. There is a notable difference in *T*_c_ determined from the electrical resistivity and the heat capacity - the 50% resistive transition precedes the midpoint of the heat capacity lambda feature by 0.6 K - but we point out that the onset of the lambda transition overlaps with the 50% resistive transition. From this, we suggest that the significant disorder in the system broadens the superconducting transition, with the bulk transition occurring near *T*_c_ ≈ 2.4 K. From the resistive transitions, an upper limit for *H*_c2_(0) is determined using the expression *H*_c2_(T) = *H*_c2_(0)(1-(*T*/*T*_c_) ^*n*^), where *H*_c2_(0) = 6.4 T and *n* = 1.27. This value of *H*_c2_(0) is similar to Pauli-paramagnetic limit for weak electron-phonon coupling, *H*_c2_(0) = 1.84*T*_c_ ≈ 5.7 K. As suggested earlier for the Zr-based analogue, this may suggest that strong spin-orbit coupling plays a role in determining the properties of the superconducting state^16^. The superconducting coherence length ζ is estimated using the expression (d*H*_c2_/dT)_Tc_ = −Φ_0_/2π*T*_c_ζ^2^, where Φ_0_ = 2.07 × 10^−7^ G/cm^2^ is the flux quantum. From (d*H*_c2_/d*T*)_Tc_ = 2.4 T/K and *T*_c_ = 3.2 K we find that ζ = 117 Å.

### Thermal properties of [TaNb]_0.31_(TiUHf)_0.69_

**Figure 4:**
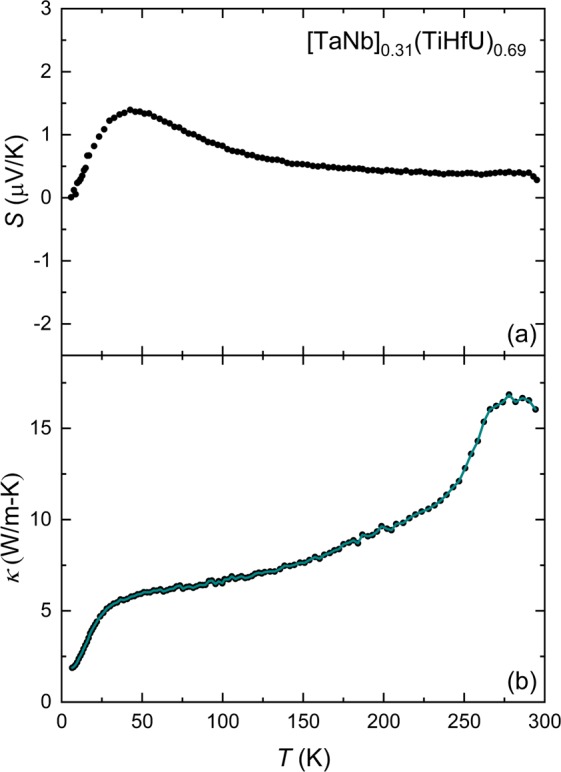
Thermal properties data for [TaNb]_0.31_(TiUHf)_0.69_. (**a**) Temperature-dependent Seebeck coefficient *S*(*T*) for [TaNb]_0.31_(TiHfU)_0.69_. (**b**) Temperature-dependent thermal conductivity *κ*(*T*) for [TaNb]_0.31_(TiHfU)_0.69_. The cyan line shows the lattice contribution to the thermal conductivity *κ*_L_, which was calculated from the expression *κ* = *κ*_E_ + *κ*_L_, where *κ*_E_ is estimated using the Wiedemann-Franz law where *κ*_E_ = L*σT* (L = 2.44 × 10^−8^ WΩK^−2^ and *σ* is the electrical conductivity determined from Fig. 2).

In order to address the thermal properties, we investigated the temperature dependent Seebeck coefficient *S*(*T*) and thermal conductivity *κ*(*T*). The Seebeck coefficient (Fig. 4a) is nearly temperature independent and exhibits small values across the entire temperature range. This indicates typical metallic behavior, similar to what is seen in other HEAs such as Y_x_CoCrFeNi, Al_x_CoCrFeNi, and (BiSbTe_1.5_Se_1.5_)_1-x_Ag_x_^32–34^. This supports our conclusion that the uranium *f*-states are delocalized and thus do not significantly impact the electrical transport behavior. At lower temperatures, *S*(*T*) exhibits a weak peak around 50 K, which we propose is due to phonon drag effect, as is seen in other HEAs due to plastic deformation at high strain rates^35–37^. The thermal conductivity (Fig. 4b) next suggests that phonon scattering mechanisms dominate the thermal transport, as would be anticipated based on the disordered arrangement of atoms on the lattice. This is seen by considering that *κ* is given by a summation of the electronic and lattice thermal conductivity *κ* = *κ*_E_ + *κ*_L_. *κ*_E_ can be estimated using the Wiedemann-Franz law where *κ*_E_ = L*σT* (L = 2.44 × 10^−8^ WΩK^−2^ and *σ* is the electrical conductivity). From this, we find that that *κ*_L_ (solid dots in Fig. 4b) dominates the total *κ* at all temperatures. Below 50 K, where phonon scattering is dominated by grain boundaries, a broad peak in *κ*(*T*) appears, which is consistent with a small phonon mean free path. At higher temperatures, *κ*(*T*) is further suppressed by both point-defect scattering and Umklapp scattering. As a result, the resulting *κ*(*T*) is quite small for a metal.

## Discussion and Conclusion

From these measurements we conclude that [TaNb]_0.31_(TiUHf)_0.69_ is a disordered type II superconductor with a bulk superconducting phase transition at *T*_c_ = 2.4 K and upper critical field *H*_c2_ = 6.4 T. Analysis of the data indicates that the superconducting state is described by the BCS theory within the weak electron-phonon coupling regime, although there is evidence that the strong spin orbit coupling impacts some of the properties. In general, these results are similar to what was earlier reported for the Zr-containing analogues, which is consistent with the conclusion that the uranium *f*-electrons are delocalized and do not (i) carry a well-defined magnetic moment or (ii) result in strong electronic correlations. Thus, it is appealing to consider the behavior of this system in the context of the framework proposed by Cava *et al*.^5,16^, where *T*_c_ for the Zr-based analogue system evolves mainly as a function of the valence electron count. For those systems, it can be estimated that [TaNb]_0.31_(TiZrHf)_0.69_ would exhibit 4.5 K < *T*_c_ < 6.5 K with an upper critical field 9 T < *H*_c2_ < 11 T, and that the Zr → U analogue behave the same way.

However*, T*_c_ and *H*_c2_ for [TaNb]_0.31_(TiUHf)_0.69_ are suppressed from the values extrapolated from the Zr analogue; i.e., the presence of the uranium ions has the effect of weakening the superconducting state. There could be several reasons for this, including that the typical effect of introducing uranium ions into conventional phonon mediated superconductors is to break Cooper pairs^19^. We also point out that earlier work in the d-electron analogues shows that there are several other important parameters that can impact the superconductivity, including the electron count and the degree of disorder. Regarding electron count, it might be anticipated that uranium would adopt a tetravalent electronic configuration. However, tetravalent uranium would be expected to produce a Curie-Weiss temperature dependence of the magnetic susceptibility and such behavior is not observed. This may indicate that the uranium ions exhibit intermediate valence behavior that quenches the local moment^17,38,39^ and that the effective electron count is smaller than anticipated. It is also possible that the introduction of an *f*-element in the place of a *d*-element, with its distinct orbitals and shape, enhances the disorder and suppresses *T*_c_. Prior work has shown that there is a positive correlation between crystallinity and the superconducting state^5^. Further work will be needed to address these questions; e.g., including X-Ray absorption spectroscopy to quantify the uranium valence and scanning tunneling electron microscopy to image the disorder.

[TaNb]_0.31_(TiUHf)_0.69_ thus emerges as an example of a small but growing family of *f*-electron materials with both practical and fundamental implications. Earlier work revealed that the Zr-based analogues and other HEAs exhibit high strength, ductility, and damage resistant properties across a broad range of temperatures, and the work presented here demonstrates that this extends to the actinide family. This suggests that this and other HEAs may provide a next generation waste form for radioactive isotopes, similar to other HEA systems^11–14^. We also anticipate that the [TaNb]_1-x_(Ti*A*Hf)_x_ HEA series will accommodate other radioactive elements (*A* = actinide) in the place of *A* = U. This has the advantage that it will enable studies of the direct impact of natural radiation damage and the long term robustness of these materials. It is also appealing to consider that variation of the stoichiometry may improve the superconducting properties and the introduction of other actinide or even lanthanide elements may induce novel behavior that might include magnetism, as has already been observed for other f-element HEAs^22–25^. This sets the stage for these systems to emerge as a next generation of novel materials with functionality along multiple axes.

## Methods

### Synthesis

Specimens were produced using the arc furnace technique, as earlier described for the Zr-based analogues^5,13,14^. The starting materials were metallic niobium (purity 99.9%), tantalum (purity 99.9%), uranium (purity 99.9%), hafnium (purity 99.9%), and titanium (purity 99.99%). The reaction took place under an argon atmosphere that was purified prior to the growth by heating a zirconium getter. After melting, the sample was rapidly cooled on a water-chilled copper plate. The sample was melted five times and turned over each time to encourage homogeneity.

### Elemental analysis

Elemental analysis was performed by scanning electron microscopy using energy dispersive spectroscopy (SEM-EDS) on an FEI NOVA 400. Samples were analyzed at an accelerating voltage of 20 kV for 60 seconds at a working distance of 10 mm. Both a large mechanically-flattened piece and small chips from the rough bottom of the boule were examined, and no statistically-significant elemental difference was observed between them.

### X-ray diffraction

Attempts at single-crystal X-Ray Diffraction (XRD) failed for even the smallest samples cut off the boule, with inherent multicrystallinity even with samples on the order of 5 μm per axis. Powder XRD was conducted on a PANalytical X’Pert PRO diffractometer with a Cu K*α* source. Extracted peaks were then assigned to d-spacings using a Microsoft Excel Spreadsheet and the Solver Tool to minimize sum-squared errors to determine the unit cell dimensions.

### Electrical resistivity, heat capacity, and magnetic measurements

DC electrical resistivity *ρ* measurements were performed in a four wire configuration for flattened specimens using a Quantum Design Physical Properties Measurement Systems with He3 option down to temperatures *T* > 0.4 K and magnetic fields *H* < 9 T. Specific heat *C* measurements were also performed in the same system using a conventional thermal relaxation technique. DC magnetic susceptibility *χ* = *M*/*H* and magnetization measurements were carried out at temperatures *T* = 1.8–300 K under an applied magnetic field of H = 5 kOe and at *T* = 1.8 K and 5 K for *H* < 7 T using a Quantum Design VSM Magnetic Property Measurement System. The samples were embedded in GE-varnish and attached to a quartz rod for the measurement. Electrical resistance *R*(*T*) measurements under applied pressure were performed using a piston cylinder pressure cell with Daphne 7575 oil as the pressure transmitting medium. The pressure was determined by the shift in ruby flourescence peaks as measured below *T* = 10 K.

### Thermopower and thermal conductivity measurements

The thermal conductivity was measured using the conventional one-heater-two-thermometer method. Two Cernox bare chip sensors were used for the thermometer and a polished thin metal chip resistor was used for the heater. A step-wise increasing heater power (d*P*) to the chip resistor generated step-wise thermal gradient across the sample. The temperature gradient (d*T*) and the corresponding thermoelectric voltage (d*V*) were simultaneously measured when d*T* was stabilized at different heater power. The thermal conductivity and the Seebeck coefficient were obtained by the relations, *κ* ~ dP/dT and *S* = d*V*/d*T*, respectively.

## Supplementary information

Supplementary Information.
