# Supplementary material for: Superconductivity in a uranium containing high entropy alloy
Source: Sci Rep. 2020 Mar 13;10:4717. doi: 10.1038/s41598-020-61666-z (PMC7070041; doi:10.1038/s41598-020-61666-z)
Supplement: Supplementary file 1 — Supplementary Information. [file 41598_2020_61666_MOESM1_ESM.docx]

**Supplementary Materials**

**Superconductivity in a uranium containing high entropy alloy**

W. L. Nelson^1,2^, A. T. Chemey^3^, M. Hertz^3^_,_ E. Choi^1^_,_ D. E. Graf^1^, S. Latturner^3^, T. E. Albrecht-Schmitt^3^, K. Wei^1^, R. E. Baumbach^1,2,*^

1. National High Magnetic Field Laboratory, Florida State University, Tallahassee, FL 32310

2. Department of Physics, Florida State University Tallahassee, FL 32306

3. Department of Chemistry, Florida State University, Tallahassee, FL 32306

Table 1: Summary of Elemental Analysis by SEM-EDS (all in atom %).

|  | Maximum | Minimum | Median | Average | Standard Deviation |
| --- | --- | --- | --- | --- | --- |
| Titanium | 27.84 | 23.22 | 24.17 | 24.53 | 1.13 |
| Niobium | 20.29 | 13.18 | 17.63 | 17.69 | 1.42 |
| Hafnium | 22.64 | 15.46 | 20.09 | 19.86 | 1.81 |
| Tantalum | 17.54 | 9.68 | 13.83 | 13.79 | 1.93 |
| Uranium | 28.23 | 20.49 | 24.15 | 24.14 | 2.24 |

Fig. S1: Electrical resistance *R* vs temperature *T* for [TaNb]_0.31_(TiUHf)_0.69_ near the superconducting transition temperature *T*_c_. Measurements were performed under hydrostatic pressure using a clamped piston cylinder cell where the pressure transmitting medium was Daphne 7575 oil.


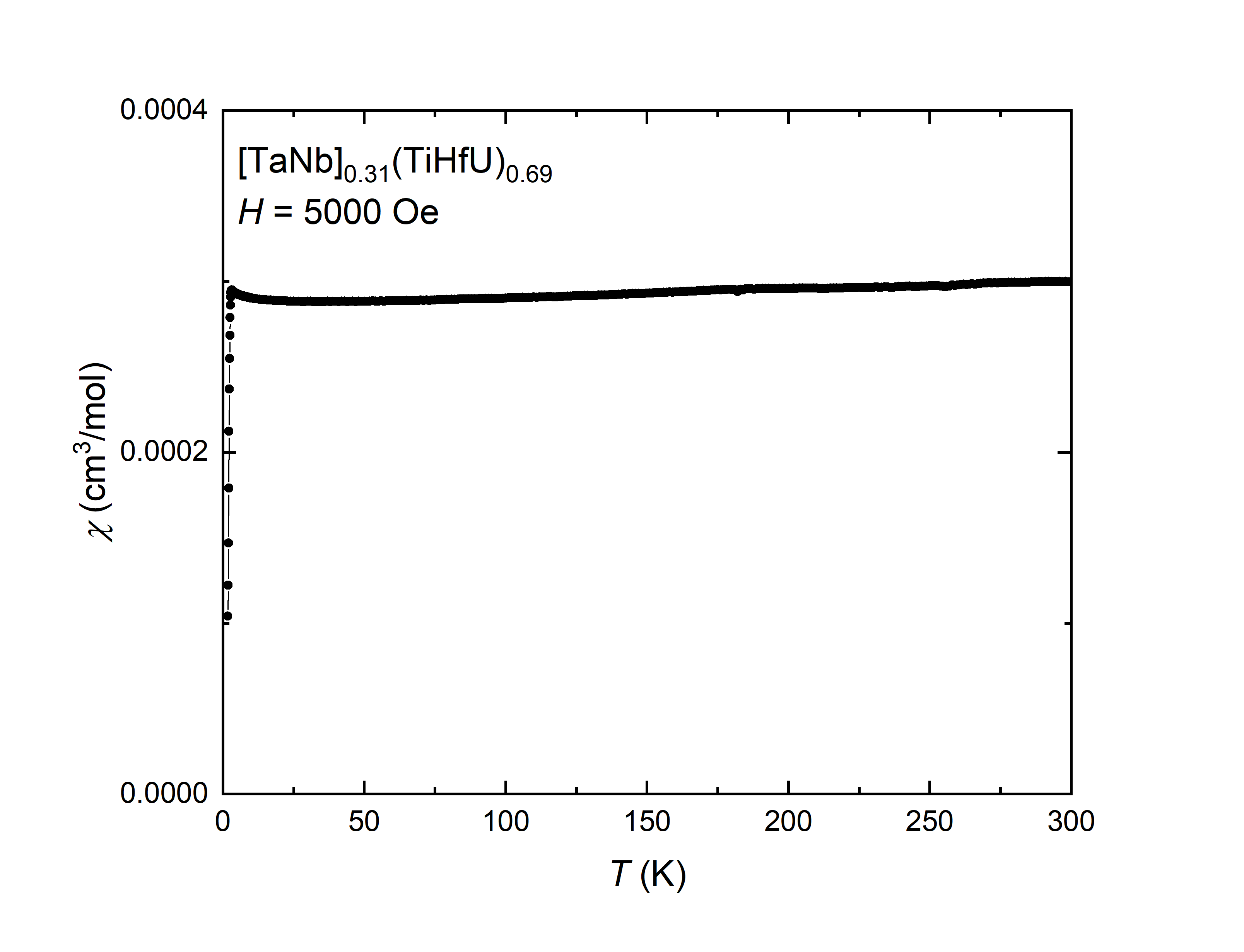


Fig. S2: The DC magnetic susceptibility *χ* = *M*/*H* vs. temperature T for [TaNb]_0.31_(TiUHf)_0.69_ collected in a magnetic field of 5000 Oe.
